# Acceptability of non-speculum clinician sampling for cervical screening in older women: A qualitative study

**DOI:** 10.1177/0969141318756452

**Published:** 2018-02-13

**Authors:** Madeleine Freeman, Jo Waller, Peter Sasieni, Anita WW Lim, Laura AV Marlow

**Affiliations:** 1 Research Department of Behavioural Science and Health, University College London, London, UK; 2 Barts & The London School of Medicine and Dentistry, Queen Mary University of London, London, UK

**Keywords:** Cervical cancer, speculum, human papillomavirus testing, postmenopause, older women

## Abstract

**Objectives:**

One reason that women over age 50 report avoiding cervical screening is increased discomfort postmenopause. This study aimed to explore the acceptability of human papillomavirus testing on clinician-collected vaginal samples without a speculum (‘non-speculum’) for cervical screening among older women.

**Methods:**

Thirty-eight women in England aged 50–64 with a range of cervical screening experience (‘up-to-date’ n = 17, ‘overdue screening’ n = 18, ‘never screened’ n = 3) were identified via a recruitment agency. Women participated in focus groups or interviews about the potential for using clinician-collected samples without a speculum. Discussions were analysed using Framework Analysis.

**Results:**

The two main themes identified were women’s perceptions of the speculum and attitudes towards non-speculum screening. Many women reported negative experiences with the speculum, including increased pain after the menopause. Women generally had positive attitudes towards non-speculum clinician sampling and felt it would be a less intrusive option, but expressed concern that it could be less accurate than screening with a speculum. Women who were ‘up-to-date’ preferred conventional screening, while overdue and never screened women welcomed the option to be screened without a speculum.

**Conclusions:**

Human papillomavirus testing on non-speculum clinician-collected vaginal samples could be an acceptable alternative cervical screening method for older women. Offering this approach could increase screening uptake in older women who find conventional cervical screening to be less acceptable with ageing or the menopause.

## Introduction

Almost half of cervical cancer deaths in the UK occur in women over age 65.^1^ Most of these occur in women who were not adequately screened when aged 50–64,^2^ yet cervical cancer screening coverage amongst women in this ‘older’ age band has been falling.^3^ Over a fifth of women aged 55–64 have not been screened in the last five years.^4^ While numerous studies have explored reasons for cervical screening non-attendance,^5–8^ few of these have explored barriers to attendance specifically in older women. A recent review of existing evidence suggests that older women cite embarrassment, an absence of symptoms, fear of pain and bad experiences (including difficulties with the smear-taker accessing the cervix), as reasons for avoiding screening.^9^ Difficulties with cervical access and discomfort may be due to vulvovaginal atrophy,^10^ which is thought to affect around half of all postmenopausal women.^11^ This could make inserting a speculum and taking a sample more painful than prior to the menopause.

Widespread dislike of the speculum has been reported among non-attenders,^6^ and almost a third of women aged 50–64 report that cervical screening has become painful with age.^12^ Given the high cervical cancer mortality rates among older women, and falling screening uptake, it has been argued that changes to the cervical screening programme should be made to accommodate the needs of older women.^13^

Human papillomavirus (HPV) causes almost all cervical cancers.^14^ HPV-based screening offers greater protection against invasive cancer than cytological screening^15^ and will be the primary cervical screening test in England from 2019.^16^ An additional benefit of HPV testing is that it enables alternative sampling methods, which could appeal to non-attenders and help increase uptake. HPV self-sampling is one such approach that has generated widespread interest. Numerous studies have shown that HPV self-sampling can increase screening uptake among non-attenders;^17^ however, women consistently worry about not taking a good-quality sample,^18^ and that samples might get contaminated or lost in the post.^19^ They may also miss the opportunity to discuss related concerns with a health professional at the time of screening.^19^ Because HPV testing on self-samples has lower specificity (for detecting high-grade cervical lesions) than clinician-taken samples (with a speculum),^20^ it is mainly considered as an approach for non-attenders.

HPV testing on clinician-collected vaginal samples taken without a speculum is another possibility. This might appeal to older women who want the reassurance of a clinician-collected sample, without the discomfort of a speculum. Unlike self-sampling, the dialogue between the woman and sample-taker would be maintained. This study assessed the acceptability of non-speculum HPV testing for cervical screening in older women, using qualitative methods.

## Methods

### Participants

Women aged 50–64 eligible for cervical screening were enrolled by an external recruitment agency, Saros (www.sarosresearch.com). Interested individuals completed an online questionnaire, which assessed their suitability for the study. Women with a previous cancer diagnosis or hysterectomy (i.e. not eligible for cervical screening) were excluded. In order to explore attitudes within specific population subgroups, women from a range of socio-economic status groups and with different cervical screening histories were sought: ‘up-to-date’ (screened within the last 5 years), ‘overdue screening’ (last screen >5 years ago) and ‘never screened’. A purposive sampling frame was determined a priori, taking into account the scope and design of the study.^21,22^ We planned to recruit two groups of ‘up-to-date’ and two groups of ‘overdue’ women. By conducting discussions with these groups separately, we hoped to encourage women who were overdue to talk more freely about their screening decisions, without fear of judgement from women who attended regularly. We anticipated that recruiting ‘never screened’ women would be more challenging, and therefore planned only one group for this screening history. The recruitment agency was commissioned to enrol eligible women into the study until our target numbers for each group were met.

### Data collection

The study was approved by the University College London (UCL) Research Ethics Committee (ref: 0496/013). Data collection took place January to February 2017. Four focus groups were conducted at UCL, each with eight women. Two groups comprised women who were ‘up-to-date’ with screening, and two comprised women who were ‘overdue screening’. The recruitment agency struggled to recruit ‘never screened’ women, and so widened their search area beyond London to the whole of England. Three women meeting the criteria were identified, and agreed to participate. As this was not considered enough to run a focus group and women were based in different areas of England, these women participated in interviews, either face-to-face (n = 1) or by telephone (n = 2). A further three women were identified as ‘never screened’ and agreed to participate in face-to-face interviews, however, once these interviews began, it became clear that these women had in fact been screened. Two of these three women were subsequently reclassified as ‘overdue screening’ and one as ‘up-to-date’.

Focus groups and interviews were moderated by MF, assisted by an additional researcher. After providing informed consent for the study, participants completed a short demographic questionnaire assessing age, marital status, work status, education, religion, ethnicity, cervical screening history (time since last cervical screening test and missed screening rounds), future cervical screening intentions and awareness of HPV. A topic guide was developed for the focus groups and interviews. This first explored previous experiences of cervical screening and the speculum examination. Two example plastic speculums (Puraspec™ in sizes small and medium) were shown to stimulate discussion about the speculum. The concept of non-speculum clinician sampling was introduced and discussed, with prompts to assess women’s concerns and perceived benefits of this method, their willingness to undertake this test and their confidence in the results. Finally, HPV self-sampling was described and an example sampler (Copan FLOQSwab™) was shown. Women were then prompted to discuss their attitudes towards this method. While attitudes to self-sampling were not the main focus of the study, self-sampling was discussed for comparison with an alternative approach to cervical screening. Open-ended questions were used throughout the focus groups and interviews, to explore attitudes in-depth. Focus groups and interviews were recorded and transcribed verbatim by a transcription company, Devon Transcription (www.devontranscription.co.uk). Focus groups lasted between 68 and 78 minutes, and interviews between 26 and 36 minutes.

### Data analysis

Data were analysed using Framework Analysis, which aims to summarize data using themed matrices, so that comparisons and contrasts may be made across and within cases (i.e. each focus group and each interview formed a case).^21^ Two researchers (MF and LM) initially read and re-read each of the transcripts to familiarize themselves with the data. They then independently coded the transcripts line by line. Next, they shared and discussed their codes. These codes were used to develop a coding framework, which aimed to capture overarching themes in the data. A matrix was created to collate examples of data which illustrated each theme. Themes were then described and explained using illustrative quotes from the data.

## Results

### Sample characteristics

Thirty-eight women took part in the study, 32 in focus groups and 6 in interviews. Table 1 shows the sample’s demographic characteristics. The mean age was 55 (range 50–64), the majority of women were White British, and half had a degree or equivalent level of education. Three quarters were employed part-time or full-time. Just over half said they had always participated regularly in cervical screening and the majority intended to attend their next cervical screening when invited. Just over half of the women had heard of HPV (54%).

**Table 1.** Participants’ demographic characteristics.

Characteristic	n = 38 (%)
Age (years)	
50–54	17 (45)
55–59	13 (34)
60–64	8 (21)
Last cervical screening	
Less than five years ago	21 (55)
More than five years ago	11 (29)
Never screened	2 (5)
Don’t know	4 (11)
Ethnicity	
White British	27 (71)
Other White background	4 (11)
Asian background	1 (3)
Black background (African/Caribbean/other black)	3 (8)
Mixed ethnic background	3 (8)
Highest level of education	
GCSEs or equivalent	8 (21)
A-level or equivalent	10 (26)
Degree or equivalent	19 (50)
Other	1 (3)
Employment status	
Employed full time	10 (26)
Employed part time	19 (50)
Unemployed	9 (24)

Numbers reflect data gathered in participant questionnaires.

The main themes, reflecting the topic guide, were women’s perceptions of the speculum and attitudes towards non-speculum testing. These themes and their subthemes are described below. Women also discussed their attitudes to HPV self-sampling and these largely reflected previous qualitative studies of HPV self-sampling. As no novel themes arose here, and this was not a primary aim of the study, we have included these findings as Supplemental material only, available online.

### Perceptions of the speculum

Women’s discussions about their perceptions of the speculum comprised two subthemes: (1) Experience of the speculum and (2) perceived benefits of the speculum.

#### Experience of the speculum

Although one of the three ‘never screened’ women had never heard of the speculum, most other women were familiar with the term ‘speculum’ and knew that it was part of the equipment used for cervical screening. However, some women, even those who were ‘up-to-date’ with screening, had never seen a speculum before and several were unaware what it was used for (i.e. to open the walls of the vagina):

P5: *I had no idea what they looked like.*

P7: *No, I had no idea. I didn’t know they opened.*

(Group 1, ‘up-to-date’)

Many women described not knowing that the speculum could be plastic and described only being familiar with the metal speculum, for which there was widespread dislike. They described it as looking ‘scary’ and ‘Victorian’, and feeling ‘cold’, ‘intrusive’ and ‘undignified’. For one of the ‘never screened’ women, the speculum was what put her off attending, following a painful failed attempt to insert it: *It was just very… very cold, this instrument, and just unable to go in and I just thought if he doesn’t stop… it was really hurting and I was telling him it was hurting but he wouldn’t stop and I then went to push his hand away because it was really hurting [followed later by]. It wasn’t an experience I wanted to repeat, so I didn’t* (Telephone interview 1, ‘never screened’).

Several women described (unprompted) how the speculum had become more painful postmenopause, and the consequences of this varied, with some saying they would be unlikely to return for screening as a result. This discussion arose in both focus groups with ‘overdue screening’ women and in one of the groups with ‘up-to-date’ women:

P8: *About five years, the doctors have wanted me to have a smear test, so I finally went about a month ago, I only let my doctor do it, female doctor because she is really nice, I didn’t want the nurse, and she just put it in and I screamed and just couldn’t do it because it was really tight and dry. So she gave me oestrogen tablets*

Mod: *Oh like a pessary. Is it a pessary?*

P8: *Yes.*

P8: *I had a tablet form, because you get a gel tablet and I wanted the tablet. But I started taking them and I thought… I just don’t want to go back to do it, it was just too painful.*

Mod: *Yes. So you haven’t been back?*

P8: *I won’t go back. She didn’t even get it like… just up to about… if you say that’s the opening, she got it that far in, I screamed. It was so painful.*

(Group 2, ‘overdue screening’)

#### Benefits of the speculum

Women perceived several benefits to using the speculum for cervical screening. These discussions about the benefits of the speculum were predominantly raised among women who were ‘up-to-date’. Many believed it was used to get a good view of the cervix and they felt reassured that the clinician could see the cervix: *The idea that occasionally they do sort of say, ‘Oh, everything looks healthy’,… even if, obviously, there’s always a possibility that the result will come back and I’ll need another one, but if it generally looks well, it’s kind of reassuring*. (P8, Group 1, ‘up-to-date’)

The speculum was also described as a way of ensuring it was easy to take the swab and that a sufficient sample could be collected quickly: ‘*It’s allowing them a full view… to see exactly where they need to go and how much they need, if they’ve taken it from the right area, and if it’s a sufficient sample being taken*’ (P4, Group 3, ‘up-to-date’). One woman described how she felt the speculum could protect the vagina while the sample was being taken: ‘*At least the one thing with that is the speculum actually is protecting your insides, and it’s actually giving them a clear passage*’ (P7, Group 4, ‘overdue screening’). Women in both of the ‘up-to-date’ groups described how the speculum was not a big issue after childbirth. This was not raised by ‘overdue screening’ or ‘never screened’ groups.

### Attitudes towards non-speculum HPV testing

Views on non-speculum HPV testing differed among women. Many women, particularly those who were ‘up-to-date’, said they would rather have the speculum used. Others, particularly in the ‘overdue screening’ group and those who had reported negative experiences with the speculum, were keen to be screened without a speculum. Four main sub-themes were identified in relation to non-speculum HPV testing: (1) Invasiveness of the sample collection, (2) time to collect the sample, (3) concerns about accuracy and (4) the need for information and reassurance.

#### Invasiveness of the sample collection

The idea of collecting a sample without using a speculum was generally described as less invasive, less intimidating and less likely to be painful than sampling with the speculum: ‘*It’s [the speculum] really painful, it opens you up, like that. That [swab], you are just putting in*’ (P8, Group 2, ‘overdue screening’).

One woman mentioned that this method would be less invasive because fewer things would be inserted into the vagina, but more women commented that the size of the sampler relative to the speculum was most important: ‘*To me, anything smaller rather bigger going in at this point is better … It would be great, I think, that there is a less intrusive way of doing it*’ (P4, Group 1, ‘up-to-date’).

Conversely, two women raised concerns about how non-speculum testing may be *more* invasive. One thought the procedure may mean the nurse had to touch you more to hold you open which would mean it was actually more invasive than having a speculum put in: ‘*If this thing is like, prising you apart slightly, are their fingers…are they going to be prising you apart with their fingers?*’ (P6, Group 3, ‘up-to-date’). A second woman worried it might be uncomfortable if the clinicians had to ‘poke about’ (Interview 2, ‘never screened’) to find the correct area.

#### Time to collect the sample

Several women anticipated that this procedure would be quicker, because the speculum was not being inserted first. Others however mentioned that it might take longer, because the sample would be more difficult to collect without the use of a speculum to see the cervix: ‘*It could take longer than a minute to try and find the right place because she would have to feel what she’s looking for because she wouldn’t be able to see, would she?*’ (Interview 2, ‘never screened’).

#### Concerns about accuracy

Several women raised concerns about the accuracy of collecting a sample without using a speculum. There were two key concerns that related to whether an ‘adequate’ sample could be collected. The first was that the swab would touch other areas of the vagina and therefore the sample would collect cells from the ‘wrong place’ by accident:

P8: *Wouldn’t it touch other things on the way to where it’s going?*

Mod: *It might do. Would that worry you?*

P8: *So picking up cells that it doesn’t want on there?*

(Group 4, ‘overdue screening’)

Secondly, women worried that the sample-taker would not be able to see the cervix without a speculum and the sample might therefore be somewhat ‘hit and miss’. The potential for a sample to be inaccurate meant a possibility of having to return for a second appointment: ‘*Would she be able to see what she’s doing?… when she sends the thing off she might not have got it in the right place and then they call you back*’ (Interview 2, ‘up-to-date’). Accuracy concerns were raised by all women regardless of screening status, but comparisons relative to cervical cytology, were limited to women who had attended screening. Among these women, there was concern that the procedure would not be as thorough as the current screening method and this led to women feel unconfident in the results. Some allayed these concerns saying that they expected clinicians would be trained in non-speculum sampling.

#### The need for information and reassurance

Women emphasised that they would want to know that non-speculum sampling was as effective as current screening, before agreeing (in theory) that they would have it done: ‘*I-I think I would want quite a bit of information and reassurance that it was gonna do the-the-the same, um, job as the-the old procedure*’ (P6, Group 1, ‘up-to-date’).

However, if non-speculum sampling was shown to be at least as effective as current practice, women were enthusiastic about this approach: *I think that as long as, like you said, it was proven to be as effective, or even more so, because generally, when people introduce change it’s for a number of good reasons. So if it was proven to be as or more effective, I would 100% go for that without hesitating, yeah*. (P4, Group 1, ‘up-to-date’)

Some women wanted to know why a ‘deep swab’ (Interview 4, ‘overdue screening’) must be taken, and why it could not just be a swab from the vaginal entrance. Others also felt they would want a choice between speculum and non-speculum sampling.

## Discussion

Overall, the findings suggest that non-speculum clinician sampling for cervical screening could be an appealing option for older women, particularly for those who may have been put off screening by the speculum examination. Women generally reported negative perceptions of the speculum, particularly increased pain on insertion since starting the menopause. They felt that non-speculum sampling could be a less intrusive alternative. However, they also raised concerns about this method, including the potential for increased invasiveness, longer time needed to take a sample, and test accuracy. These issues would need to be addressed in information materials on non-speculum sampling methods. We do not believe that these concerns would persist if the correct information were provided, but there would clearly be a need to educate practice nurses and GPs about the benefits and limitations of this form of cervical screening if it were to be introduced, so that accurate information can be passed on to patients.

To our knowledge, this is the first study to explore women’s attitudes towards non-speculum clinician-sampling for cervical screening. Perspectives were sought from women from a range of screening backgrounds in order to compare differences in attitudes and assess whether this method might promote uptake in women who are overdue (or have never attended) for cervical screening. The sample was diverse in terms of education level and employment status, so the findings represent a range of views we might expect among this age group more generally. Another strength is the focus specifically on older women, an age group for which data are sparse, yet cervical cancer mortality rates (in women aged 65+) are high.^1^

We did not provide detailed information about HPV itself, so discussion was limited to the sample collection methods only. This meant that women were not told that they would most likely have to return for a speculum examination if they tested positive for HPV, nor that the sampling method of HPV testing is likely to influence test accuracy (for detecting cervical disease). Nor were they told that it was not necessary to visualize, or sample from, the cervix to obtain a good sample for HPV testing. It is therefore likely that providing women with more information to allay these concerns might have resulted in even more positive views on the non-speculum method.

As with other studies exploring barriers to cervical screening, women who had never attended screening were difficult to recruit.^5,23^ The challenges recruiting women who have never been screened may be in part because numbers of never screened women among those aged 50–64 (excluding those ceased for clinical reasons) are very low (around 3%^4^). It is also possible that women who have never been screened were less interested in participating in research on a topic of which they have no experience.

Our finding that some women find insertion of the speculum to be more painful since starting the menopause, and that this has deterred some from attending screening, supports research showing that a third of women aged over 50 have found cervical screening painful with age.^12^ Although use of oestrogen creams/pessaries can relieve discomfort associated with vaginal atrophy,^24^ our study demonstrates that for some older women, there is a strong preference for alternative methods of screening, which do not require a speculum. Importantly, the preference for non-speculum clinician-sampling was mostly amongst women overdue for screening, while women who were up-to-date preferred conventional screening. This implies that offering non-speculum sampling would enhance and not undermine the current screening programme.

Women’s concerns about non-speculum sampling are similar to those found by acceptability studies for HPV self-sampling.^25^ This highlights the importance of producing information materials for non-speculum clinician sampling, which address concerns about test accuracy and clearly explains test procedures (including invasiveness and time). Widespread concern amongst older women about not taking a good sample for self-sampling, and the logistics of returning the kit (as reported in the supporting information), suggests that non-speculum clinician sampling could be an additional (and potentially more appealing) option to women who would prefer the reassurance of having a clinician take the sample.

## Conclusion

HPV testing on clinician-collected vaginal samples without a speculum could be an acceptable method of cervical screening for older women among whom speculum examination is a barrier to screening. Clear descriptions of the tests and procedures involved will be critical to allay concerns about these alternative screening methods. Further research into the accuracy of testing using such samples is warranted.

## Supplementary Material

Supplementary material
