# Supplementary material for: Acceptability of non-speculum clinician sampling for cervical screening in older women: A qualitative study
Source: J Med Screen. 2018 Feb 13;25(4):205–10. doi: 10.1177/0969141318756452 (PMC6262594; doi:10.1177/0969141318756452)
Supplement: Supplementary material [file supplementary_material.pdf]

<u>Attitudes to HPV self-sampling</u>

Women were generally positive about HPV self-sampling. Women who were 'overdue screening' or 'never screened' were particularly enthusiastic, with all 'never screened' women saying they would be willing to try it. Five subthemes were identified: 1) Practical benefits, 2) Control, 3) Logistical concerns, 4) Concerns about accuracy and 5) Confidence.

Practical benefits: Women recognised several practical benefits to HPV self-sampling, some which would directly benefit them and others which would benefit the wider public. Direct benefits related to time, both taking less time: "*it's the time factor as well. At least you can make the time to do that at some point in your day"* (P6, Group 2, 'overdue screening'), and having the freedom to choose the test timing: "*at your own time and pace. Not anybody else's"* (Telephone interview 2, 'never screened'). Women also mentioned how self-sampling would save resources: "It doesn't take up the doctor's time, or the nurse's time" (Telephone interview 1, 'never screened'). Several women also pointed out that they thought HPV self-sampling would reduce costs: "*These probably cost tuppence to produce, um, do it at home and you've... you know. They saved themselves a lot of money"* (P3, Group 1, 'up-to-date').

Control: Women emphasised that HPV self-sampling would enable them to be in control: *"I would definitely do that myself. Because you are in control, aren't you, you are in complete control"* (Telephone interview 1, 'never screened'). As a result of this control, women felt this method would be less painful: "*it wouldn't be uncomfortable because you would be in control of it, you would be doing it yourself"* (Telephone interview 1, 'never screened').

Logistical concerns: Women expressed several logistical concerns about HPV self-sampling. These included women not doing the test or not returning it, and the sample validity: *"When they do this swab, how many days are allowed to reach the person who is going to check it? How many days grace? People might swab but just keep it there and they don't… They will leave it on the table, oh I'll take it to the GP or I'll post it, I'll post it, and they never do"* (Interview 3, 'up-to-date'). Issues surrounding posting samples were also raised, including concern about contamination and samples getting lost in the post:

P5: *What about stuff that gets lost in the post as well?*
P7: *Oh, yes. Your cells get lost in the post.*
P5: *Can you imagine you'd done it yourself, or whatever, and then you sit there, and you just never get a result because it never actually arrived?* (Group 3, 'up-to-date')

Concerns about accuracy: Many of the women were concerned about the accuracy of HPV self-sampling. This generally related to false-negatives: *"I'd be worried that you would be doing it, and then say they had some cells and they were bad and then next to it the ones were alright and you took them from the bit that was alright"* (P6, Group 2, 'overdue screening'). Women also worried that they might perform the test incorrectly and have to re-do it:

P8: *But with this, I think there'd be a lot of recalls, though.*
P1: *So do I.*
P8: *I don't think people would do it properly.*
P1: *I wouldn't do it properly.*
P8: *They'd be too scared to really put it... Because you have to really put it inside, haven't you?* (Group 3, 'up-to-date')

One woman who had never been screened also mentioned that although she was "frightened" of being told she has abnormal cells after cervical screening, she would feel less anxious about her test result if she did it herself, because she would assume human error if it came back positive: *"if I got*

called back, abnormal cells, I'd think oh I've done it wrong. I wouldn't worry as much if they called me back" (Interview 2, 'never screened').

Confidence to do the test: Some women suggested a lack of confidence in their ability to self-sample, e.g.: *"I would feel a little bit unsure about whether I... I would probably lack confidence as to whether I was doing it properly."* (P8, Group 1, 'up to date'). One woman felt that offering self-testing could be counter-productive because more time would be spent answering questions than would have been required for a clinician to collect a sample. Another woman said she'd prefer a nurse to do the self-test for her: *"if the nurse was there and she was putting this thing inside of me then I would feel more comfortable."* (P3, Group 2, 'overdue screening').

Other women pointed out that women who have limited mobility may be unable to do the test themselves: "*It does beg the question... that as you get older and less dextrous, whether you are able to do it yourself"* (P3, Group 1, 'up-to-date'). Several women mentioned that women who have never used tampons might also struggle with self-sampling, not only physically but psychologically: "*I know that psychologically they might not feel comfortable – 'Oh there's something down my thing. I've never done it myself', so maybe a bit panicky or nervous about doing it."* (Interview 3, 'up-to-date').

Conclusions

Women generally had positive attitudes towards HPV self-sampling, particularly those who were 'overdue screening' and 'never screened'. The main benefits related to practicality and control. Concerns over accuracy, ability and logistics were raised – consistent with previous findings. Overall, the findings suggest HPV self-sampling for cervical screening could be an appealing option for some older women who may have been put off screening by the speculum examination.
